# A retrospective study of factors associated with treatment decision for nontuberculous mycobacterial lung disease in adults without altered systemic immunity

**DOI:** 10.1186/s12879-018-3559-x

**Published:** 2018-12-14

**Authors:** Judith Provoost, Florent Valour, Delphine Gamondes, Sandrine Roux, Nathalie Freymond, Emilie Perrot, Pierre-Jean Souquet, Lize Kiakouama-Maleka, Christian Chidiac, Gérard Lina, Oana Dumitrescu, Agathe Sénéchal, Florence Ader

**Affiliations:** 1 Département de Pneumologie, Hospices Civils de Lyon, Lyon, France; 2 Département des Maladies infectieuses et tropicales, Hôpital de la Croix-Rousse, Hospices Civils de Lyon, 103, Grande-Rue de la Croix-Rousse, 69317, cedex 04 Lyon, France; 3 CIRI-Centre International de Recherche en Infectiologie, Inserm, U1111, Université Claude Bernard Lyon 1, CNRS, UMR5308, Ecole Normale Supérieure de Lyon, Univ Lyon, F-69007 Lyon, France; 4 Université Claude Bernard Lyon 1, Lyon, France; 5 Département de Radiologie, Hospices Civils de Lyon, Lyon, France; 6 Institut des Agents Infectieux, Hospices Civils de Lyon, Lyon, France

**Keywords:** *Aspergillus* spp., Bronchiectasis, Chronic obstructive pulmonary disease, Hemoptysis, Mycobacteria, *Mycobacterium avium complex*, Nontuberculous mycobacteria

## Abstract

**Background:**

Nontuberculous mycobacteria (NTM) lung diseases are increasingly recognized as chronic opportunistic infections, occurring in individuals with a wide variety of underlying conditions. In the absence of systemic immunodeficiency, decision of NTM lung disease treatment must relies on a careful risk/benefit assessment, given the requirement of long-term administration of multidrug therapies supported by limited evidence. The primary objective was to identify the factors associated with anti-NTM treatment initiation. Clinical and radiological outcome upon treatment were studied.

**Methods:**

This retrospective, single center study (2013–2016, 45 months) addressed the criteria supporting treatment decision among adults with NTM lung disease without systemic immunodeficiency at our institution, with the assigned goal to harmonize the practice. All patients matched the current international definitions of NTM lung disease according to the American Thoracic Society criteria. Factors associated with anti-NTM treatment were investigated by conditional logistic regression. Clinical and radiological outcomes of treated and untreated NTM-disease cases were examined. Mortality rate was assessed. An expert radiologist conducted a blinded computed tomography (CT)-scan review of the treated and untreated patients.

**Results:**

Among 51 cases of NTM lung diseases, 25 (49%) received anti-NTM treatment. In univariate analysis, a body mass index (BMI) < 18 kg/m^2^ (odds ratio (OR), 4.2 [95% confidence interval (CI) 1.2–15.2]; *p* = 0.042), hemoptysis (OR, 11.8 [95% CI 1.35–12.9]; *p* = 0.026), excavation(s) (OR, 4.8 [95% CI 1.4–16.4], *p* = 0.012), prior anti-NTM treatment (OR, 5.65 [95% CI 1.06–29.9]; *p* = 0.042), *Aspergillus* spp. co-infection (OR, 6.3 [95% CI 1.8–22.2]; *p* = 0.004) were associated with treatment initiation. In multivariate analysis, *Aspergillus* spp. co-infection was the only independent determinant of treatment initiation (OR, 5.3 [95% CI 1.1–25.4]; *p* = 0.036). Twenty-one (81%) patients received ≥3 anti-NTM drugs. Median treatment duration and follow-up were 36.3 (interquartile range [IQR], 13.1–64.4) weeks and 17.1 (IQR, 8.7–27.1) months, respectively. Regarding radiological outcome, 85 CT-scans were reviewed, showing similar rates of regression or stabilization in treated and untreated patients. Overall mortality rate was not different in treated and untreated patients.

**Conclusion:**

The most relevant variable associated with anti-NTM treatment initiation was *Aspergillus* spp. co-infection. Radiological regression or stabilization of pulmonary lesions was not different between the treated and untreated patients.

## Background

Nontuberculous mycobacteria (NTM) are ubiquitous bacteria of environmental origin including a widely diverse number of species (> 160), some of which cause disease in humans [1–3]. Prevalence of NTM lung diseases is unexpectedly increasing in industrialized countries, as consistently uncovered by recent studies [3, 4]. The key issue remains to determine whether NTM are the true and single promoter of an evolving lung disease or chronic airway colonizers, among others. To standardize the diagnosis of NTM lung disease, the guidelines for NTM diagnosis of the American Thoracic Society (ATS)/Infectious Disease Society of America (IDSA) and the British Thoracic Society (BTS) require isolation and growth of the same NTM strain on at least two separate samples from the patient [5, 6].

Human host and pathogenic NTM relationship is still poorly understood, as NTM virulence is highly variable from one species to another. NTM lung disease is strongly associated with pre-existing pulmonary conditions such as chronic obstructive pulmonary disease (COPD), cystic fibrosis, idiopathic bronchiectasis, prior active tuberculosis or pneumoconiosis [6]. It is also frequently associated with genetic or acquired systemic immune deficiency such as defects in the pathways of inflammatory cytokines interleukin (IL)-12, tumor necrosis factor (TNF)-α or interferon (IFN)-γ, immunosuppressive treatments (including anti-TNF-α therapy or corticosteroids), solid-organ transplantation, or acquired immune deficiency syndrome (AIDS)/human immunodeficiency virus (HIV) infection [1–7]. However, it may also occur in individuals without recognized severe immune local or systemic deficiency. In the absence of patent predisposition, NTM diseases are overrepresented among the specific morphotype of slender women with a low body fat [8].

Treatment decision for NTM lung disease is challenging. There is debate as to which patients should benefit the most from treatment according to medical background, comorbidities, clinical status, radiologic features and causal NTM strain. Assessment of clinical, microbiologic, and radiologic response to treatment is not standardized as well. We focused on NTM lung diseases in adults without systemic immunodeficiency that met the ATS criteria guidelines. Based on the comparison of a group of treated and untreated patients, the primary objective was to identify the factors associated with physician decision of initiating anti-NTM treatment. Secondary objectives were to study the outcome upon treatment and to propose a standardized evaluation for the diagnosis and decision making to treatment of NTM lung diseases in adults without systemic immunodeficiency.

## Methods

### Study design and patient population

We conducted a retrospective, observational, single-center study between January 2013 and February 2016 (45 months) among adults (≥ 18 year-old) without systemic immunosuppression presenting NTM lung disease. Exclusion criteria were HIV infection, cystic fibrosis, primary ciliary dyskinesia, active malignant disease, solid-organ transplantation or ongoing immunosuppressant treatments such as TNF inhibitor or high-dose corticosteroid (≥ 1 mg/kg more than 21 days). Case identification was based on cross-referencing the databases of the mycobacteria laboratory and the departments of infectious and pulmonary diseases. Patients eligible for inclusion in the NTM lung disease cohort were those who matched the criteria previously defined by the ATS/IDSA and the BTS guidelines with the minimum requirement of clinical and microbiologic following criteria: *(i)* pulmonary symptoms associated with multifocal bronchiectasis with multiple small nodules on computed tomography (CT)-scan; *(ii)* proper exclusion of other diagnoses; *(iii)* NTM-positive culture results from at least two separate expectorated sputum samples or a NTM-positive culture result from at least one bronchial wash or lavage [5, 6]. Patients’ characteristics at diagnosis were collected in order to perform analysis on 146 selected variables: demographics; history of predisposing factors; underlying pulmonary diseases; comorbidities; pulmonary function testing; respiratory bacterial or mycological co-infection(s), which definition was similar to NTM criteria, namely positive culture isolation of the same species from at least two separate expectorated sputum samples or a positive culture result from at least one bronchial wash or lavage; immunologic status; nutritional status; clinical features; microbiologic assessment through identification of NTM species on positive NTM cultures and sample culture conversions; radiologic features on high-resolution CT-scans (fibrocavitary disease or nodular/bronchiectasis disease); prior treatment for NTM lung disease, treatment combination and duration; outcome. Because of the retrospective observational nature of the study and the lack of any modification in patients’ management, the need for informed consent was waived with the authorization of the Ethics Committee of Lyon University Hospital (Comité d’Ethique, Hospices Civils de Lyon), which approved the study under the number 17–207.

### Radiologic assessment

An independent expert chest radiologist, blinded to the patient information, retrospectively reviewed the CT-scans performed without injection of intravenous contrast media, assigned in random order at diagnosis and six to 24 months after treatment or during the follow up of the untreated patients. The number and size of cavity(ies) and their wall thickness were evaluated in the lung window setting. Nodular opacity(ies) (≥ 10 mm), cluster(s) of small nodules (≤ 5 mm), the tree-in-bud pattern, the presence of bronchiectasis in any of the lobes or multifocal bronchiectasis were evaluated. Based on the number and size of the lesions, the expert classified the lesions as improved, stable or worsening.

### Endpoints

The primary endpoint was to identify the factors significantly involved in the decision of initiating anti-NTM treatment by patient referent physician. Secondary endpoints were the assessment of clinical and radiological outcomes upon anti-NTM treatment in comparison with no treatment. Based on these findings, a standardized appraisal was proposed to assist diagnosis management and treatment decision for NTM lung diseases in adults without altered systemic immunity.

### Statistical analysis

Descriptive data were used to estimate the frequencies of the study variables. There were expressed as count (percentage, %) for dichotomous variables and as medians (interquartile range [IQR]) for continuous values. The number of missing values was excluded from the denominator. Non-parametric statistical methods Fisher exact test, χ2 test and Mann-Whitney U test were used to compare groups, where appropriate. The probability of treatment initiation over time was evaluated by Kaplan-Meier survival curve analysis, with group comparison using the log-rank (Mantel-Cox) test. Stepwise binary logistic regression analysis was used to assess the determinants for treatment initiation, expressed as odd ratios (OR) with 95% confidence intervals (95% CI). After checking the variables for interactions, variables with medical meaning and with *p*-values obtained in the univariate analysis of < 0.15 were included in the final multivariate model. A value of *p* < 0.05 was considered significant. All analyses were performed using SPSS software version 24.0 (SPSS. Chicago. IL).

## Results

**Fig. 1:**
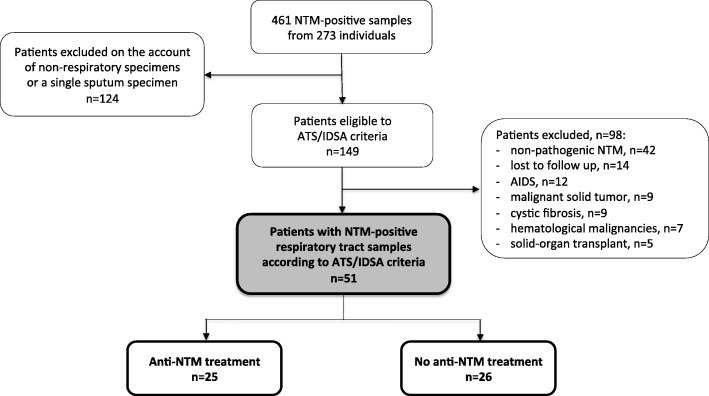
Flow chart of adult patients with nontuberculous mycobacterial lung disease without altered systemic immunity included in the study. Nontuberculous mycobacterial lung disease defined according to the American Thoracic Society/Infectious Diseases Society of America and the British Thoracic Society [4, 5]

Out of 149 patients eligible to ATS/IDSA NTM lung disease criteria, 51 patients were included in the study, of who 25 (49%) received and 26 (51%) did not receive anti-NTM treatment (Fig. 1). The median age was 68 (interquartile range [IQR], 59–75) year-old with a male/female ratio of 0.76. Etiologic NTM agents were *Mycobacterium avium* (*n* = 17, 33.3%), *M. chimaerae* (*n* = 14, 27.5%), *M. xenopii* (*n* = 9, 17.6%), *M. intracellulare* (*n* = 9, 17.6%), *M. simiae* (*n* = 3, 5.9%), *M. kansasii* (*n* = 1, 2%), and *M. abscessus* (*n* = 1, 2%), with three patients having ≥2 concomitant NTM lung diseases. Importantly, the evidence of NTM lung disease has led to diagnose six underlying chronic lung diseases, of which a genetically documented cystic fibrosis in a 61 year-old women. On descriptive analysis, patient’s characteristics did not significantly differ, to the exception of a lower BMI (*p* = 0.023) and a higher number of previously known NTM lung disease (*p* = 0.038) in treated versus untreated patients (Table 1). Notable percentages of missing data at diagnosis have to be acknowledged for active tobacco smoking (64.7%, *n* = 33), respiratory functional testing (23.5%, *n* = 12) with a very few patients having a 6-min walk test, baseline arterial blood oxygenation levels (58.8%, *n* = 30), CT-scan follow up within 24 months after diagnosis in untreated patients (46%, *n* = 12).

**Table 1:** Patients’ demographic and baseline descriptive characteristics

	Treated patients (*n*=25)	Untreated patients (*n*=26)	*P*-value
Age, median (IQR) years	65 (54-74)	70 (60.8-76)	0.227
Gender (male)	9 (36)	13 (50)	0.4
Active smokers	6 (24)	6 (23)	1
Cumulative tobacco smoking (IQR) pack/year	32.5 (28.8-52.5)	40 (21.3-60)	0.79
BMI (IQR) kg/m^2^	17.9 (16.8-19.9)	19.9 (18.3-23.8)	***0.023***
History of tuberculosis	6 (24)	5 (19.2)	0.74
History of NTM lung disease	8 (32)	2 (7.7)	***0.038***
History of croup during infancy	1	1	1
Gastroesophageal reflux disease	4 (16)	2 (7.7)	0.42
History of cured malignant solid tumor	1 (4)	5 (19.2)	0.19
Diabetes mellitus	0	2 (7.7)	0.49
Chronic heart failure	0	4 (16)	0.11
Asthma or atopic disease	6 (24)	2 (7.7)	0.14
Chronic obstructive pulmonary disease	4 (16)	9 (34.6)	0.19
Bronchiectasis	14 (56)	14 (53.8)	1
Chronic interstitial lung disease	2 (8)	5 (19.2)	0.42
Chronic rheumatic disease	6 (24)	2 (7.7)	0.14
Autoimmune disease	3 (12)	4 (15.4)	1
History of thoracic surgery	5 (20)	3 (11.5)	0.46
Corticosteroid inhaled therapy	6 (24)	7 (27)	1

NOTE Data are No. (%) of patients, unless otherwise indicated. Variables were compared using Mann–Whitney U test, χ2 test or the Fisher exact test, where appropriate

Abbreviations: IQR, interquartile range

Factors leading the patient referent physician to initiate anti-NTM treatment were assessed using bivariate analysis. Among the factors significantly associated with treatment were the BMI < 18 kg/m^2^ (odds ratio (OR), 4.2 [95% confidence interval (CI) 1.2–15.2]; *p* = 0.042), prior anti-NTM treatment (OR, 5.65 [95% CI 1.06–29.9]; *p* = 0.042), hemoptysis (OR = 11.8 [95% CI 1.35–102.9]; *p* = 0.026), the presence of excavation(s) on lung CT-scan (OR, 4.8 [95% CI 1.4–16.4], *p* = 0.012), *Aspergillus* spp. co-infection (OR, 6.3 [95% CI 1.8–22.2]; *p* = 0.004) (Table 2). In multivariate analysis, *Aspergillus* spp. co-infection was the single independent factor associated with anti-NTM treatment (OR, 5.3 [95% CI 1.1–25.4]; *p* = 0.036) (Table 3).

**Table 2:** Bivariate analysis of the factors associated with treatment initiation in adult patients with nontuberculous mycobacterial lung disease without systemic immunodeficiency

Tested variables	Treated patients (n=25)	Untreated patients (n=26)		*P-value*	OR [95% CI]
*Clinical criteria*					
BMI < 18 (kg/m^2^)	13/24 (54.2)	5/23 (21.7)		***0.026***	4.25 [1.18-15.2]
Weight loss > 5%	17 (68)	10 (38.5)		***0.05***	3.4 [1.07-10.77]
fatigue	13 (52)	7 (27)		0.071	2.94 [0.9-9.46]
chronic cough	16 (64)	14 (54)		0.572	1.52 [0.49-4.69]
night sweats	5 (20)	3 (11.5)		0.465	1.92 [0.41-9.05]
hemoptysis	8 (32)	1 (3.8)		***0.026***	11.8 [1.3-102.8]
*Microbiological criteria*					
AFB-positive sputum sample on direct examination	13 (52)	8 (30.8)		0.160	2.44 [0.77-7.65]
NTM-positive culture NTM on lower respiratory tract sample	2 (1-2.3)	1 (1-1.8)		***0.044***	**1.79 [1-3.2]**
*Functional criteria*					
FEV1 (L)	1.6 (1.2-2.2)	1.9 (1.2-2.5)		0.554	0.72 [0.34-1.51]
*Immunological and nutritional status*					
total lymphocyte count (G/L)	1.3 (1.2-1.7)	1.4 (1.1-2.1)		0.388	0.95 [0.86-1.04]
CD4+ lymphocyte count (%)	54 (4.5-61.5)	40 (37.8-44.3)		***0.031***	**1.07 [0.95-1.21]**
serum albumin (mg/L)	39.5 (36-43)	39 (33-42.5)		0.481	1.05 [0.97-1.14]
serum pre-albumin (mg/L)	0.21 (0.15-0.24)	0.22 (0.15-0.25)		0.974	1.25 [0-1.48]
vitamin D deficiency	6/10 (60)	6/7 (86)		0.338	0.25 [0.02-2.95]
*Co-infection(s)*					
*Mycobacterium tuberculosis*	1 (4)	1 (4)		0.35	1.04 [0.06-17.6]
*Pseudomonas* spp.	2 (8)	3 (11.5)		1	0.68 [0.1-4.4]
*Aspergillus* spp.	15 (60)	5 (19.2)		***0.004***	**6.3 [1.8-22.2]**
*Radiologic features at diagnosis*					
nodule(s) (≥ 10mm)	12 (48)	7/23 (30)		0.25	2.11 [0.65-6.9]
cluster(s) of micronodules (< 5mm)	16 (64)	10/23 (44)		0.246	2.31 [0.72-7.4]
cavitation(s)	18 (72)	8/23 (35)		***0.019***	**4.82 [1.42-16.4]**
bronchiectasis	14 (56)	11/23 (48)		0.773	1.39 [0.45-4.3]
emphysema	9 (36)	11/23 (48)		0.559	0.61 [0.19-1.95]
*Radiologic review*					
regression	8/23 (35)	4/13 (31)		1	1.2 [0.28-5.15]
stabilization	9/23 (39)	6/13 (46)		0.736	0.75 [0.19-2.97]
worsening	6/23 (26)	3/13 (23)		1	1.18 [0.24-5.8]

NOTE Data are No. (%) of patients, unless otherwise indicated. Denominators have been stipulated for variables with missing data. Odds ratios (OR) were determined by conditional logistic regression with 95% confidence interval (95% CI)

Abbreviations: AFB, acid-fast bacilli; BMI, body mass index; CI, confidence interval; FEV1, forced expiratory volume in 1 second; NTM, nontuberculous mycobacteria; OR, odds ratio

**Table 3:** Multivariate analysis of factors associated with the initiation of anti-nontuberculous mycobacteria treatment

Independent variables	OR [95% CI]	*P*-value
*Aspergillus* spp.	***5.33 [1.1-25.5]***	***0.036***
weight loss > 5%	2.44 [0.6-10.2]	0.22
hemoptysis	3.2 [0.28-36.3]	0.35
excavation(s)	3.54 [0.83-15.1]	0.087

Abbreviations: CI, confidence interval; OR, odds ratio

**Fig. 2:**
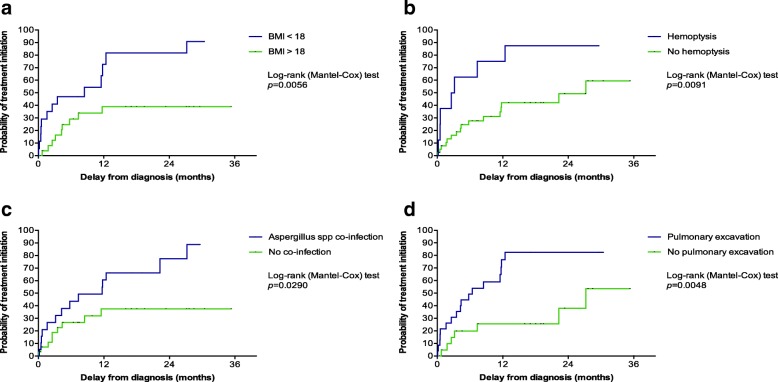
Overall probability of anti-nontuberculous mycobacteria treatment initiation according to the presence or the absence of the following variables: **a** body mass index < 18 kg/m^2^; **b** hemoptysis; **c** *Aspergillus* spp. co-infection; **d** lung excavation(s). Overall survival probabilities were determined by the Kaplan-Meier method and were compared by the log-rank test

The probability of treatment initiation over time according to the presence or not of targeted variables was investigated. The probability was significantly higher in case of BMI < 18 vs. > 18 kg/m^2^ (*p* = 0.006), of hemoptysis vs. no hemoptysis (*p* = 0.009), of *Aspergillus* spp. co-infection vs. no co-infection (*p* = 0.029), of pulmonary excavation(s) vs. no excavation (*p* = 0.005) (Fig. 2a, b, c and d, respectively). In the treated group, 21 (84%) received ≥3 anti-NTM drugs, among whom 23 (92%) received a macrolide as part of a first-line regimen. Median anti-NTM treatment duration was 36.3 (IQR, 13.1–64.4) weeks. Thirteen (52%) of the treated patients developed at least one antimicrobial-related adverse event, among which gastrointestinal disorders (*n* = 6), impaired blood cell count (*n* = 5) and cholestatic and/or cytolytic hepatitis (*n* = 4). According to physician judgment, clinical stabilization and improvement was obtained for 8 (34.8%) and 12 (52.2%) of treated patients, respectively. Patient’s median follow-up from diagnosis assessment was 17.1 (IQR, 8.7–27.1) months. Eighty-five CT-scans were reviewed for comparative assessment after treatment or over the course of follow-up in the untreated subset. The median interval between the initiation of NTM treatment and the date of the assessed images was 56.7 (IQR, 43.3–97.7) weeks. Although 34.8% (*n* = 8) and 39.1% (*n* = 9) experienced regression or stabilization of their pulmonary lesions in the treated patients, these rates did not differ from the untreated patients with 30.8% (*n* = 4) and 46.2% (*n* = 6) of regression and stabilization over the course of their follow-up, respectively (Table 2). Finally, all-cause mortality was not different between treated and untreated groups, although lost to follow-up was high in the untreated group (*n* = 10, 38.5%). Regarding the four patients that deceased (*n* = 3 in the treated group and *n* = 1 in the untreated group), the cause of mortality was linked to the underlying diseases rather than NTM-related mortality.

## Discussion

In the present study, treated patients were characterized by BMI < 18 kg/m^2^, presence of hemoptysis and excavation(s), *Aspergillus* spp. co-infection, and prior anti-NTM treatment. *Aspergillus* spp. co-infection was the only independent factor associated with treatment initiation. A single study from five English centers has recently addressed the factors that influence anti-NTM treatment initiation using similar retrospective design of treated and untreated cohort comparison (2010–2014), to the exception of different inclusion criteria which allowed non-AIDS/HIV immunosuppressed patients (36 and 33% in the treated and untreated subsets, respectively) to be evaluated. In the multivariate analysis, patients had increased odds of anti-NTM treatment in case of cavitation on CT imaging, night sweats and weight loss [9].

Here, three out of four criteria are part of guideline criteria leading to decision of anti-NTM treatment. They all reflect a degree of severity of NTM lung disease linked with progression of an impaired respiratory condition. The current problematic of NTM lung diseases shifts from distinguishing colonization from infection toward differentiating stable, poorly active vs. progressive active NTM disease, the latter being responsible for further structural lung damage(s). The risk/benefit analysis includes prescribing recommended long-term multidrug regimens with concerns over suboptimal cure rates and frequently reported drug-related side effects. To support these arguments, others have already shown that physician ATS/IDSA guideline adherence is moderate confirming such a trend of intuitive clinical behavior mostly based on evidence of severity and/or patent progression of the disease. A multicenter study has reported a sample of 349 US physicians treating 915 patients with *M. avium* complex (MAC) lung disease. Overall, 55% (*n* = 411) of patients with MAC lung disease received some type of anti-NTM treatment from their physician and 13% prescribed antibiotic regimens were consistent with ATS/IDSA guidelines [10]. Another study conducted in France has shown that among a cohort of 31 NTM lung diseases, only 12 (38.7%) received appropriate treatment matching ATS/IDSA guidelines [11]. Inappropriate prescriptions were mostly related to shorter treatment duration (6 months or less) and/or off-recommendation regimen, particularly those excluding macrolide from the combination or those using a single-drug macrolide regimen [10, 11]. It has to be acknowledged that guidelines specify that treatment for MAC-associated lung disease in HIV-negative individuals can be a three-times-weekly drug regimen upon culture conversion while on therapy for 1 year, which may favor treatment compliance [5, 12, 13].

Patients with bronchiectasis and NTM lung disease have a higher prevalence of being sensitized to *Aspergillus* than patients with NTM-free bronchiectasis [12]. Allergic airway manifestations in response to *Aspergillus* are termed *Aspergillus*-related lung diseases with a spectrum going from *Aspergillus*-induced hypersensitivity to the severe allergic bronchopulmonary aspergillosis (ABPA) [14]. By itself, the coexistence of NTM and *Aspergillus* in lung airway justifies the need for testing *Aspergillus* serology, total serum immunoglobulin (Ig)E and *Aspergillus*-specific IgE levels as well as mycological direct examination and culture of sputum or bronchial aspirates for presence of filamentous fungi in the diagnosis algorithm of patients with NTM lung diseases. Active co-infections with NTM and *Aspergillus* spp. have also been described, in which patients with NTM lung disease develop chronic forms of pulmonary aspergillosis which definitions and management have been revisited in recent updated guidelines [14–17].

Radiographic improvement may be hampered by concomitant lung disease and the limited potential for resolution of consolidated radiologic abnormalities. A previous study has investigated radiologic response to treatment showing consistent results with those found in the present study. Although anti-NTM treatment led to an improvement or stabilization of lesions for a majority of patients, these modifications were not significantly different from the untreated group who went through CT-scan follow up indicating that anti-NTM treatment did not lead to radiological abnormalities reversion [9]. The present study has strengths and limitations. The strength is the study of the largest cohort so far of NTM lung diseases in patients without systemic immunodeficiency with exhaustive data collection and blinded radiological assessment. We acknowledge the biases that contribute to mitigate conclusions from the study such as being conducted in a single center, the important differences in physician’s management resulting in lack of consistency in treatment decision making, the number of missing data. In addition, the CT-scans were not performed at fixed intervals, particularly in the untreated subset of patients. Finally, treatment duration and outcome criteria were not standardized, which prevent to properly assess treatment efficacy. Useful consensus definitions for key outcome parameters to be used in the treatment of NTM lung diseases have been released very recently, which should harmonize data collection regarding NTM treatment [18].

Future researches are necessary to better define criteria associated with progressive active NTM disease in the immunocompetent setting. Concretely, this preliminary study has led to implement in our institution a standardized appraisal for the diagnosis of NTM lung diseases in this particular setting (Table 4). The aim is to provide a future basis for the development of a diagnosis scoring system supporting anti-NTM treatment decision. Future studies should focus as well on the most relevant CT imaging variables associated with response to treatment over time that may be applied in future clinical trials to assess treatment outcome.

**Table 4:** Standardized appraisal for patients with nontuberculous mycobacterial lung diseases without systemic immunodeficiency referred at our institution

	Comments
*Exhaustive history record*	
infant respiratory diseases	croup, Influenza, measle
gastro-oesophageal reflux disease	
atopic dermatitis	
eating disorders	anorexia, bulimia
family history of respiratory disease	
prior tuberculosis	
prior anti-NTM treatment	
*Environmental exposure*	
passive tobacco exposure	
active tobacco smocking	Packs-per-year
other toxic/drug exposure	
chronic alcoholism	micro-inhalations
habitat characteristics	domestic water system, allergens
*Blood tests*	
serum albumin	nutritional status
serum pre-albumin	
vitamin D (25-OH D3)	
HIV serology	cellular immune defect
T CD4+ and CD8+ lymphocyte counts	
total IgG serum level	hypogammaglobulinemia
total eosinophil count	*Aspergillus*-related allergic lung diseases
total IgE serum level	
*Aspergillus*-specific IgE	
*Aspergillus* serology	
galactomannan antigenemia	invasive aspergillosis
Interferon-γ reactive assay	latent tuberculosis infection
*Lung function testing*	
spirometry including FEV1	lung volumes
plethysmography	
pulse oxymetry	oxygen level
arterial blood gas tests	
6-minutes walk test	stress test
*Imagery*	
chest X-ray	
lung HRCT scan	Micronodules (≤ 5mm), nodules (≥ 10 mm), pulmonary consolidations, bronchiectasis, tree-in-bud images, cavity(ies)
*Cytology and microbiologic tests on respiratory samples*	
BALF cytological composition	
sputum/BALF direct examination	
sputum/BALF collection for bacterial culture	Non-fermenting gram-negative bacilli (*Pseudomonas aeruginosa*)
sputum/BALF collection for mycological culture	filamentous fungi
nasal swab/BALF collection for respiratory virus PCR screening	human Respiratory syncytial virus, parainfluenza virus, metapneumovirus, rhinovirus, and coronavirus
*Specific tests depending on the setting*	
α-1 antitrypsin level	lung emphysema
sweat test	cystic fibrosis
CFTR gene screening	
airway cilia sample	primary ciliary dyskinesia
IFN-γ and IL-12 plasma levels	host predisposition to NTM diseases
blood anti-IFN-γ autoantibodies	
IFN-γ or IL-12 gene screening	

Abbreviations: BALF, bronchoalveolar lavage fluid; CFTR, cystic fibrosis transmembrane conductance regulator, FEV1, forced expiratory volume in 1 second; HRCT, high-resolution computed tomography; IFN-γ, interferon gamma; Ig, immunoglobulins; IL, interleukin

## Conclusions

In summary, the main factors supporting anti-NTM treatment decision in immunocompetent were low BMI, hemoptysis, lung excavation(s), prior anti-NTM treatment and *Aspergillus* pp. co-infection, the latter being the only independent factor. Anti-NTM treatment did not achieve radiological abnormalities reversion, as pulmonary lesions assessment showed no difference between the treated and the untreated patients. A diagnosis of NTM lung disease in an immunocompetent patient requires investigating the presence of a chronic pulmonary underlying disease.

## Abbreviations

AIDS: Acquired immune deficiency syndrome
ATS: American thoracic society
BTS: British thoracic society
COPD: Chronic obstructive pulmonary disease
CT: Computed tomography
HIV: Human immunodeficiency virus
IDSA: Infectious disease society of America
IFN: Interferon
IL: Interleukin
NTM: Non tuberculous mycobacteria
TNF: Tumor necrosis factor
